# Asperuloside inhibits the activation of pancreatic cancer-associated fibroblasts via activating transcription factor 6

**DOI:** 10.1007/s12672-024-01095-w

**Published:** 2024-06-19

**Authors:** Ling-zhi Cao, Fan-hui Yang, Hao Zhang, Ai-min Jia, Su-ping Li, Hu-ling Wen

**Affiliations:** 1 Department of Nuclear Medicine, The Affiliated Hospital of North Sichuan Medical College, Nanchong, 637000 Sichuan People’s Republic of China; 2 Institute of Rheumatology and Immunology, The Affiliated Hospital of North Sichuan Medical College, Nanchong, 637000 Sichuan People’s Republic of China; 3 Department of Nuclear Medicine, Cancer Hospital of Shantou University Medical College, Shantou, 515041 Guangdong People’s Republic of China

**Keywords:** Asperuloside, Activating transcription factor 6, Cancer-associated fibroblasts, Normal fibroblasts, Pancreatic cancer

## Abstract

**Background:**

Pancreatic cancer-associated fibroblasts (CAFs) play a crucial role in tumor progression and immune evasion. Asperuloside (ASP) is an iridoid glycoside with potential anti-tumor properties. This study aimed to explore the molecular mechanisms of ASP on CAFs, particularly focusing on its effects on activating transcription factor 6 (ATF6), a key regulator of endoplasmic reticulum stress.

**Method:**

CAFs were treated with different concentrations of ASP (0, 1, 3, and 5 mM), and the role of ATF6 was investigated by over-expressing it in CAFs. Subsequently, western blot was used to detect ATF6, α-smooth muscle actin (α-SMA), fibroblast activating protein (FAP), and vimentin protein levels in CAFs. The collagen gel contraction assay and Transwell assay were applied to evaluate the contraction and migration ability of CAFs. In addition, the interleukin (IL)-6, C–C motif chemokine ligand (CCL)-2, and C-X-C motif chemokine ligand (CXCL)-10 levels were detected by reverse transcription-quantitative polymerase chain reaction (RT-qPCR).

**Results:**

CAFs had significantly higher expression levels of α-SMA, FAP, and vimentin compared to normal fibroblasts (NFs). ASP significantly inhibited the activation, contraction, and migration of CAFs in a concentration-dependent manner. ASP treatment also reduced the expression of cytokines (IL-6, CCL2, and CXCL10) and down-regulated ATF6 levels. Over-expression of ATF6 mitigated the inhibitory effects of ASP.

**Conclusion:**

ASP exerts its anti-tumor effects by down-regulating ATF6, thereby inhibiting the activation and function of pancreatic CAFs. These findings suggest that ASP could be a promising therapeutic agent for pancreatic cancer by modulating the tumor microenvironment.

**Supplementary Information:**

The online version contains supplementary material available at 10.1007/s12672-024-01095-w.

## Background

Pancreatic cancer is an invasive malignancy and the main cause of cancer-related death worldwide [1, 2]. Although great progress has been made in the diagnosis and treatment for cancers in the past decade, the prognosis of pancreatic cancer is still poor, and the 5-year survival rate of patients is only 9% [1]. Even in patients with early diagnosis and surgical resection, the 5-year survival rate is still only 34% [3]. Up to now, there has been no effective treatment for pancreatic cancer. Therefore, it is very important to find and develop effective treatment methods to improve the prognosis of pancreatic cancer patients.

Tumor microenvironment plays an important role in influencing cancer progression and prognosis. To be specific, tumor cells can interact with the surrounding complex stromal cell ecosystem to form a tumor microenvironment [4]. Tumor microenvironment is mainly composed of extracellular matrix (ECM), cancer-associated fibroblasts (CAFs), lymphocytes, and vascular endothelial cells [5]. Among them, CAFs are the most prominent and key cellular components in the microenvironment [4]. There is evidence that CAFs can produce dense fibrosis or hyperplastic connective tissue within and around tumors to promote the malignant biological behavior of cancer cells [6, 7]. In addition, activated CAFs can regulate the immune response by secreting cytokines such as interleukin (IL)-6 and chemokines such as C–C motif chemokine ligand (CCL) 2 and C-X-C motif chemokine ligand (CXCL) 10, and then help cancer cells escape immune surveillance [8, 9]. Therefore, targeting CAFs presents a promising therapeutic strategy for pancreatic cancer, given their crucial role in modulating the tumor microenvironment and promoting cancer progression through mechanisms such as cytokine and chemokine secretion, extracellular matrix remodeling, and immune evasion.

Activating transcription factor 6 (ATF6) is one of the key stress sensors on the endoplasmic reticulum (ER) [10, 11]. There are several studies that ATF6 is related to the occurrence and progression of various cancers. For instance, ATF6 could regulate cell growth and migration, inhibit cell apoptosis and autophagy via ER stress or mitogen-activated protein kinase (MAPK) pathway [12]. Besides, inhibiting nuclear factor erythroid 2-related factor 2 (Nrf2)-ATF6 pathway can block the activation of CAFs and inhibit the progression of lung cancer [13]. The up-regulation of ATF6 was related to the worse recurrence-free survival (RFS), overall survival (OS), and disease-specific survival (DSS) of pancreatic cancer patients; in addition, ATF6 promoted the proliferation and invasion of pancreatic cancer cells [14]. However, there is no report on the effect and related mechanism of ATF6 on pancreatic CAFs.

Chemotherapy and radiotherapy are important components of current cancer therapy, but they are associated with high toxicity and side effects, which impair the survival rate of patients. A large number of studies have reported the anti-tumor potential of chemicals and derivatives from plants [15, 16]. Asperuloside (ASP) is an iridoid glycoside mainly isolated from plants belonging to the *Rubiaceae* or *Eucommiaceae* family. ASP has many pharmacological effects, including anti-tumor, anti-inflammation, and anti-oxidation [17]. Some studies have reported the inhibitory effect of ASP on tumors. ASP can promote the apoptosis of cervical cancer cells Hela and CaSki through the ER stress-mitochondrial pathway [18]. ASP inhibited epithelial-mesenchymal transition (EMT) by regulating the vitamin D receptor (VDR)/Smad3 pathway, thereby preventing the occurrence of colorectal cancer [19]. However, the effect of ASP on pancreatic cancer remains to be clarified.

Hence, this study aimed to investigate the effects and mechanisms of ASP on pancreatic CAFs [20]. The main objectives were to (1) evaluate the inhibitory effects of ASP on the activation of pancreatic CAFs, (2) assess the impact of ASP on the contraction and migration abilities of CAFs, (3) determine the influence of ASP on cytokine and chemokine expression levels in CAFs, and (4) explore whether the effects of ASP on CAFs are mediated through the down-regulation of ATF6. Overall, our research provided a new theoretical basis for the application of ASP in the treatment of pancreatic cancer.

## Materials and methods

### Chemicals and reagents

**Fig. 1:**
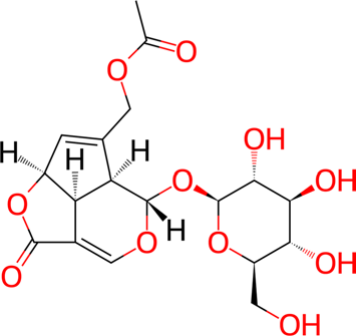
The chemical structure of ASP (Drawn by chemical draw software, version 20)

Dulbecco’s Modified Eagle Medium (DMEM) was from Invitrogen, USA. Fetal bovine serum (FBS), penicillin, and streptomycin were purchased from Gibco, USA. Asperuloside (ASP, CAS#14259-45-1, HPLC ≥ 98%) (Fig. 1) was obtained from Yuanye, China. Radio immunoprecipitation assay (RIPA) lysis buffer, 1% protease inhibitor, phosphorylase inhibitor, 5% bovine serum albumin (BSA), Tris-buffered saline with Tween (TBST), phosphate-buffered saline (PBS), and 0.1% crystal violet were purchased from Beyotime Biotechnology, China. Lipofectamine 2000 (11668-019), BCA protein assay kit (A53226), and Trizol were provided by Thermo Fisher Scientific, USA. The PrimeScript RT kit was purchased from Takara, Japan, and the SYBR GREEN kit from Yeasen, China. Cell matrix type I-A was purchased from Nitta Gelatin, Japan.

### Cell culture and treatment

#### Cell culture and identification

CAFs and normal fibroblasts (NFs) were purchased from SAIOS Biotechnology Co., Ltd (Wuhan, China), which were originated from pancreatic cancer tissue and normal pancreatic tissue, respectively. The cells were cultured in the Dulbecco’s Modified Eagle Medium (DMEM, Invitrogen, Carlsbad, CA, USA) supplemented with 10% fetal bovine serum (FBS), 2% penicillin and streptomycin under the condition of 5% CO_2_ and 37 °C. After that, the cell morphology was observed by an electron microscope (ECLIPSE E100, Nikon, Japan) and the images were collected. Further, western blot was used to detect the expression of fibroblast-specific markers α-smooth muscle actin (α-SMA), fibroblast activating protein 1 (FAP-1), and vimentin in CAFs and NFs.

#### Cell viability assay

Cell viability was measured by the CCK-8 assay [20]. After inoculation of CAFs and NFs in 96-well plates at a density of 4000 cells per well for 24 h, the supernatant was removed and a complete medium with different ASP concentrations was added. After further incubation for 24 h, the supernatant was aspirated and complete medium containing 10% CCK8 was added, and continued to incubate protected from light for 1 h. The absorbance at 450 nm was then detected in a microplate reader (ELx800, BioTek, USA).

#### Cell transfection

CAFs in the logarithmic growth phase were collected, digested, and seeded into cell culture dishes. When cell confluence reached 60–70%, transfection was performed. The pcDNA3.1-ATF6 (ATF6 over-expression plasmid) and the negative control pcDNA3.1-vector plasmid were designed and synthesized by BGI Genomics (China). These plasmids were transfected into CAFs using Lipofectamine 2000 (11668-019, Thermo Fisher Scientific, Rockford, IL, USA) according to the manufacturer’s instructions, ensuring efficient gene delivery and expression.

#### Experimental grouping

To explore the effect of different concentrations of ASP on the activation of CAFs, CAFs were divided tinto four groups: 0 mM group: CAFs were treated with 0 mM of ASP; 1 mM group: CAFs were treated with 1 mM of ASP; 3 mM group: CAFs were treated with 3 mM of ASP; 5 mM group: CAFs were treated with 5 mM of ASP. To clarify the role of ATF6, CAFs were divided into four groups: Control group: CAFs were not subjected to any treatment; ASP group: CAFs were treated with ASP (5 mM) for 24 h; ASP + vector group: CAFs were treated with ASP (5 mM) for 24 h after transfection with pcDNA3.1-vector; ASP + ATF6 group: CAFs were treated with ASP (5 mM) for 24 h after transfection with pcDNA3.1-ATF6 [21].

#### Western blot

The cells were lysed with radio immunoprecipitation assay (RIPA) lysis buffer (P0013B, Beyotime Biotechnology, Shanghai, China) containing 1% protease inhibitor and phosphorylase inhibitor. After centrifugation, the supernatant was collected. Next, a bicinchoninic acid (BCA) protein assay kit (A53226, Thermo Fisher Scientific, Rockford, IL, USA) was used to determine the protein concentration. After that, the total protein was mixed with 5 × loading buffer (P1040, Solarbio, Beijing), heated for 10 min, and denatured. Subsequently, the protein samples were separated by sodium dodecyl sulfate–polyacrylamide gel electrophoresis (SDS-PAGE) and transferred to the polyvinylidene fluoride (PVDF) membrane. The PVDF membrane was cut into sections prior to hybridization with antibodies to focus on specific protein regions of interest.Then, the membrane was sealed with 5% bovine serum albumin (BSA) at room temperature. Upon 1 h, the membrane was incubated overnight at 4 ℃ with primary antibodies as follows: [FAP (1: 1000, ab207178, Abcam), vimentin (1: 1000, ab92547, Abcam), α-SMA (1: 1000, ab7817, Abcam), ATF6 (1:1000, ab37149, Abcam) and glyceraldehyde-3-phosphate dehydrogenase (GAPDH, 1: 1000, ab8245, Abcam). Subsequently, the membrane was washed with TBST, and the second antibody (1: 5000, ab205718/ab205719, Abcam) was added for another 1 h of incubation at room temperature. After that, the chemiluminescent liquid (WBAVDCH01, Sigma, MO, USA) was dripped evenly on the membrane, and an imager (Amersham Imager 600, USA) was employed for scanning and collecting images. Besides, Image J (version 1.8.0, NIH, USA) was used for gray- level analysis. Finally, data were normalized using GAPDH as a reference, and the relative expression of target proteins was quantified.

#### Reverse transcription-quantitative polymerase chain reaction

Total RNA was extracted from each group of cells using Trizol (Thermo Fisher Scientific, USA) and the concentration was quantified through a NanoDrop 2000c spectrophotometer. The extracted total RNA was reversely transcribed into cDNA using the PrimeScript RT kit (Takara, Japan). Next, cDNA was used to detect the expression of target genes according to the instructions of the SYBR GREEN kit (Yeasen, China) on the Thermal Cycler Dice^®^ Real-Time System. The procedure was conducted using a Real-time fluorescence quantifier (Bio-rad, USA) and data was analyzed using the 2^−ΔΔCt^ method with GAPDH as the internal control gene. The primer sequences are shown in Table 1.

**Table 1:** The primer sequences of reverse transcription-quantitative polymerase chain reaction

Genes	Primer sequences
IL-6	F 5’-AGACAGCCACTCACCTCTTC-3’
	R 5’-TTTCACCAGGCAAGTCTCCT-3’
CCL2	F 5’-CTCATAGCAGCCACCTTCAT-3’
	R 5’-TCCTGAACCCACTTCTGCTT-3’
CXCL10	F 5’-GCCATTCTGATTTGCTGCCT-3’
	R 5’-GCAATGATCTCAACACGTGGA-3’
GAPDH	F 5’-ATGGGTGTGAACCACGAGA-3’
	R 5’-CAGGGATGATGTTCTGGGCA-3’

#### Collagen gel contraction assay

A collagen gel contraction assay was performed to check the contraction of cells [22]. In brief, cells (3 × 10^5^) were mixed with 1 ml cell matrix type I-A (Nitta Gelatin, Osaka, Japan) and seeded in 12-well plates. The gel mixture was incubated at 37 °C for 30 min to polymerize the gel. Then, 1 mL serum-free medium was added, and the gel was separated from the well wall with the pipette tip. After incubation for 48 h, the wells were imaged. Subsequently, Image J software (version 1.8.0, NIH, USA) was utilized to measure the gel surface area and calculate the gel contraction percentage [the gel contraction percentage = (gel surface area/well surface area) × 100%].

#### Transwell assay

Firstly, 100 μL cell suspension (1 × 10^5^ cells/mL) was seeded into Transwell chambers, and the lower chamber was supplemented with 500 μL DMEM containing 10% FBS. After 24 h of culture, the Transwell chamber was taken out and washed twice with phosphate buffered saline (PBS). Then, the cells on the upper chamber surface were wiped off with a cotton swab. After methanol fixation for 30 min, the cells were stained using 0.1% crystal violet for 20 min. Finally, the cells were observed by an inverted microscope (ECLIPSE E100, Nikon, Japan), and five fields of view were randomly selected to photograph and count.

### Statistical treatment

SPSS21.0 (IBM SPSS Statistics, Chicago, IL, USA) was used for statistical analysis. The data were expressed by mean ± standard deviation (SD). An independent sample t- test was used for comparison between two groups, and one-way analysis of variance (ANOVA) for comparison among multiple groups. *P* < 0.05 was considered as the criterion for evaluating the statistically significant difference.

## Results

### Identification of the fibroblasts

**Fig. 2:**
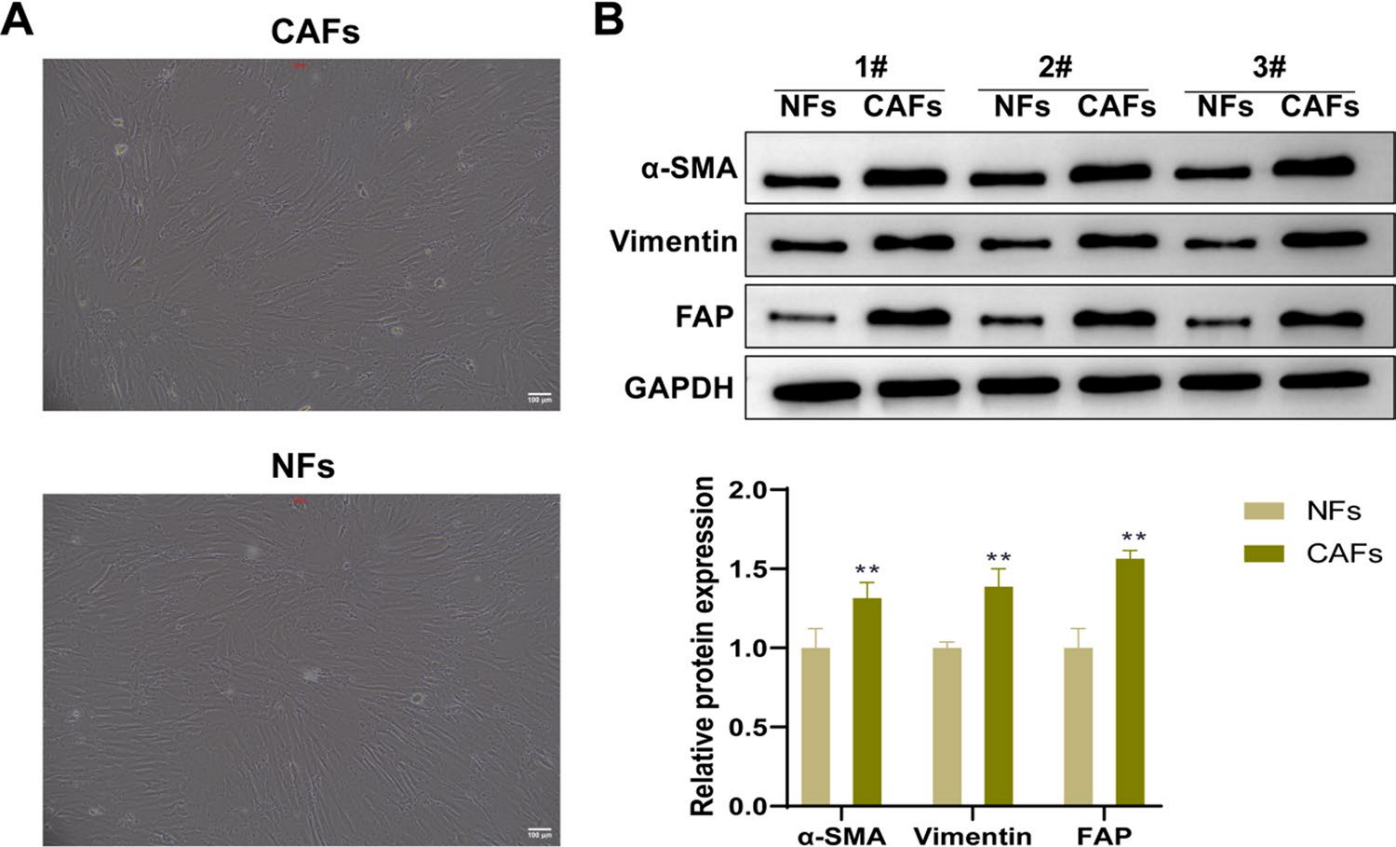
Identification of the fibroblasts. **A** The morphology of fibroblasts (CAFs and NFs) was observed under the microscope. Scale bar = 100 μm; **B** The levels of α-SMA, FAP and vimentin in CAFs and NFs were detected by western blot. The data were expressed as SEM ± mean. n = 3, **P < 0.01 vs. NFs. *CAFs* cancer-associated fibroblasts, *NFs* normal fibroblasts, *α-SMA* α-smooth muscle actin, *FAP* fibroblast activating protein, *SEM* standard error of the mean

First, we observed the cell morphology of CAFs and NFs. Under the microscope, NFs and CAFs were spindle-shaped (Fig. 2A). Subsequently, we detected the expression of cancer-related fibroblast-specific markers α-SMA, vimentin and FAP in NFs and CAFs. The results showed that the protein expression levels of α-SMA, vimentin, and FAP in CAFs were significantly higher than those in NFs (*P* < 0.01, Fig. 2B).

### Asperuloside treatment inhibits the activation and cytokine expression of cancer-associated fibroblasts

**Fig. 3:**
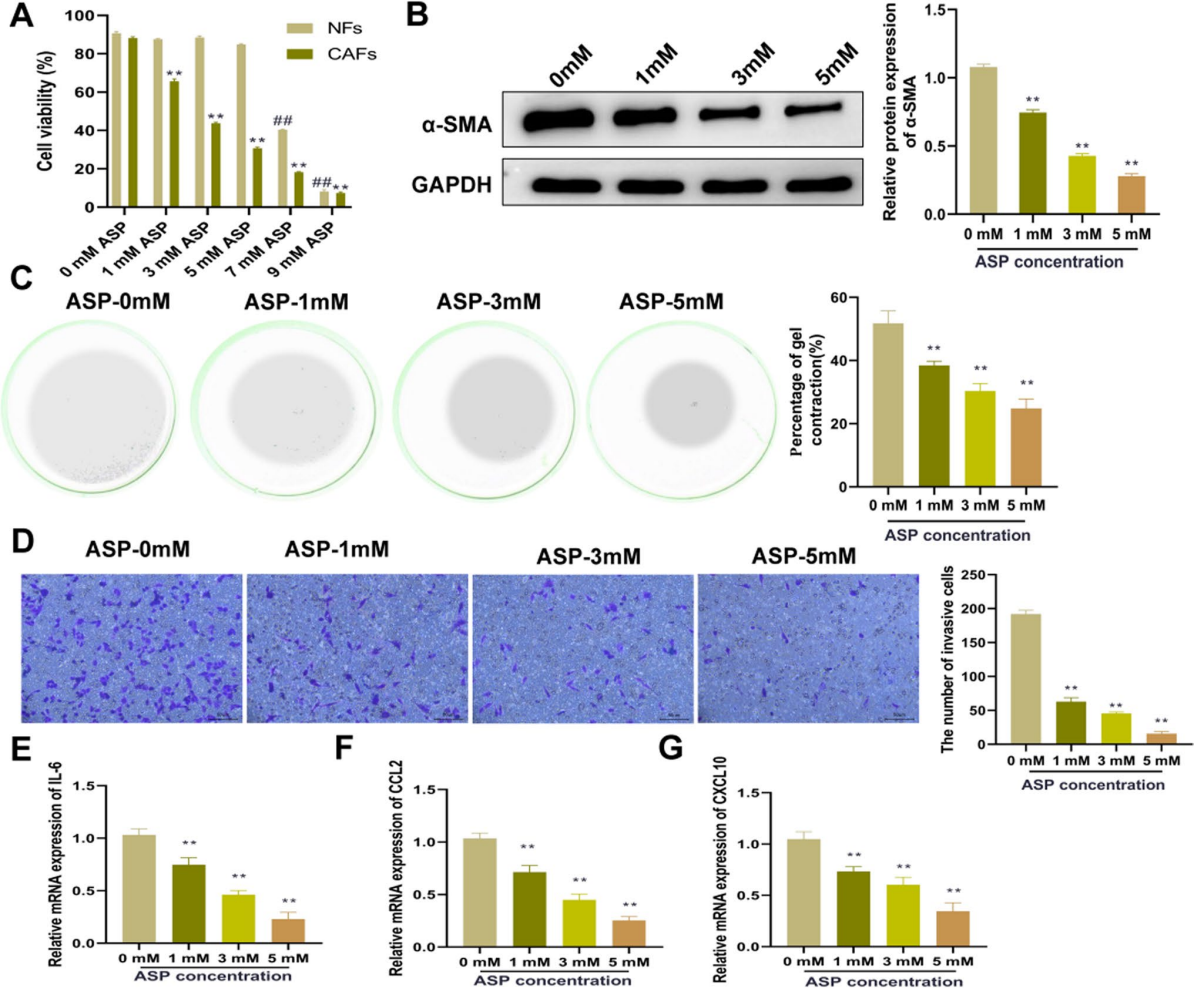
Asperuloside inhibits the activation and cytokine expression of cancer-associated fibroblasts. **A** CCK-8 assay was used to detect the effect of ASP on the cell viability of NFs and CAFs. **P < 0.01 vs. 0 mM (CAFs), ## *P* < 0.01 vs. 0 mM (NFs). **B** Western blot was used to detect the effect of ASP on the protein expression level of α-SMA in CAFs; **C** The effect of ASP on the contraction of CAFs was determined by collagen gel contraction experiment; **D** Transwell was employed to examine the influence of ASP on the migration ability of CAFs; **F**–**H** RT-qPCR was adopted to check the effect of ASP on the mRNA expression levels of *IL-6* (**E**), *CCL2* (**F**) and *CXCL10* (**G**) in CAFs. n = 3, **P < 0.01 vs. 0 mM. *ASP* asperuloside, *α-SMA* α-smooth muscle actin, *CAFs* cancer-associated fibroblasts, *RT-qPCR* reverse transcription-quantitative polymerase chain reaction, *IL-6* interleukin-6 *CCL2* C–C motif chemokine ligand 2, *CXCL10* C-X-C motif chemokine ligand 10

To explore the cell toxicity of ASP, NFs and CAFs were intervened with different concentrations of ASP, the CCK-8 result showed that ASP significantly reduces the viability of CAFs in a dose-dependent manner, while having minimal impact on NFs (Fig. 3A). However, the cell viability of NFs significantly declined at 7 mM and 9 mM ASP (*P* < 0.01), thus, we selected 1 mM, 3 mM and 5 mM ASP for subsequent experiments. Next, the changes in α-SMA expression in CAFs were assessed. The results of the western blot showed that ASP significantly reduced the protein expression level of α-SMA in CAFs in a concentration-dependent manner (*P* < 0.01, Fig. 3B). Next, collagen gel contraction assay and Transwell were applied to evaluate the effect of ASP on the contraction and migration ability of CAFs. The results showed that ASP inhibited the contraction and migration ability of CAFs, and the inhibition effect of 5 mM ASP was the most significant (*P* < 0.01, Fig. 3C, D). In addition, we also found that ASP inhibited the expression levels of cytokines (*IL-6, CCL2*, and *CXCL10*) in CAFs in a concentration-dependent manner (*P* < 0.01) (Fig. 3E–G). The above results indicated that ASP inhibited the activation and cytokine expression of CAFs in a concentration-dependent manner.

### Asperuloside treatment down-regulates the expression of activating transcription factor 6 in cancer-associated fibroblasts

**Fig. 4:**
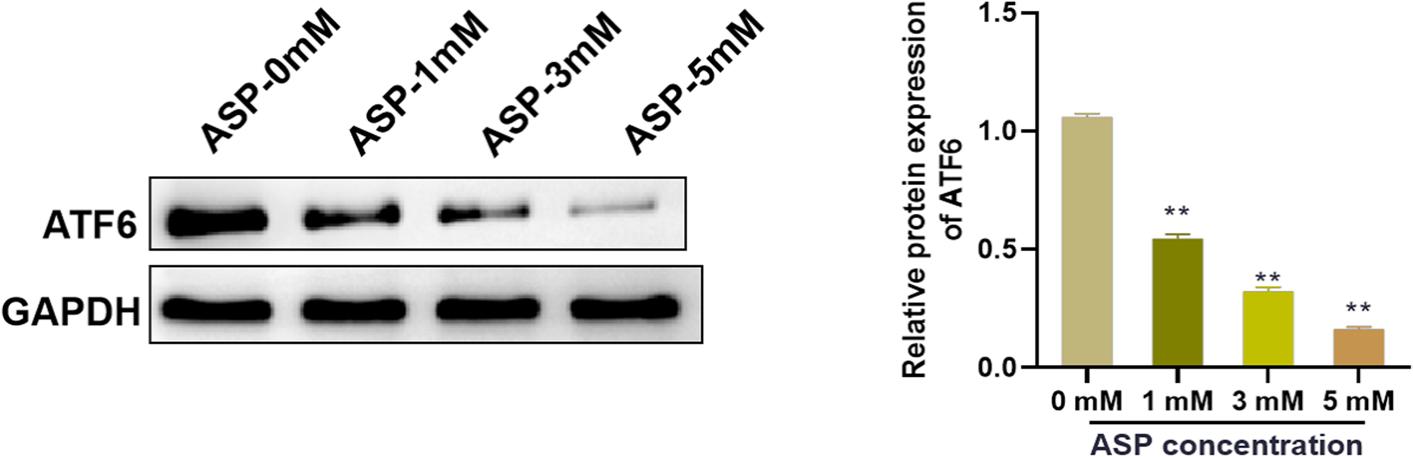
Asperuloside treatment down-regulates the expression of activating transcription factor 6 in cancer-associated fibroblasts. Western blot was adopted to detect the effect of ASP on the expression level of ATF6 protein in CAFs. n = 3, **P < 0.01 vs. 0 mM. *ASP* asperuloside, *ATF6* activating transcription factor 6, *CAFs* cancer-associated fibroblasts

To further explore the molecular mechanism of ASP affecting CAFs, the protein expression changes of ATF6 were detected. Western blot analysis showed that ASP treatment reduced the expression level of ATF6 protein in CAFs in a concentration-dependent manner (Fig. 4). Therefore, ATF6 may be involved in the inhibitory mechanism of ASP on CAFs.

### Up-regulation of activating transcription factor 6 reverses the inhibitory effect of asperuloside on the activation of cancer-associated fibroblasts

**Fig. 5:**
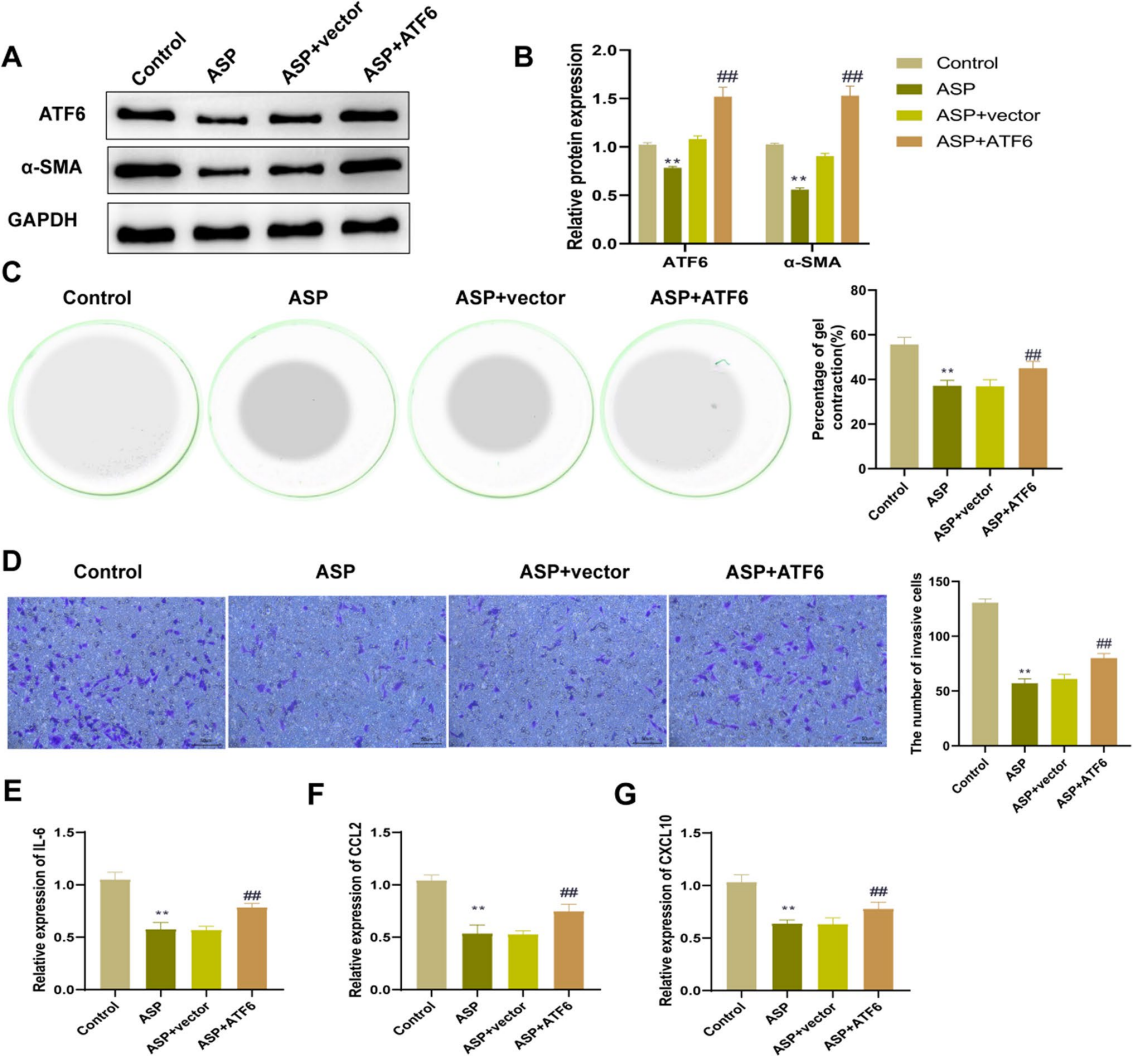
Up-regulation of activating transcription factor 6 reverses the inhibitory effect of asperuloside on the activation of cancer-associated fibroblasts. **A**, **B** The protein expression levels of ATF6 and α-SMA in cells of each group were detected by western blot; **C** Collagen gel contraction test was used to evaluate the contraction of cells in each group; **D **The migration ability of cells in each group was detected by Transwell experiment; **F**–**H** The mRNA expression levels of *IL-6* (**E**), *CCL2* (**F**) and *CXCL10* (**G**) in cells of each group were measured by RT-qPCR. *ATF6* activating transcription factor 6, *α-SMA* α-smooth muscle actin, *IL-6* interleukin-6, *CCL2* C–C motif chemokine ligand 2, *CXCL10* C-X-C motif chemokine ligand 10, *RT-qPCR* reverse transcription-quantitative polymerase chain reaction. n = 3, **P < 0.01 vs. Control group; ##*P* < 0.01 vs. ASP + vector group

To further verify the role of ATF6 in inhibition of CAF activation by ASP, CAF cells over-expressing ATF6 were constructed by transfection with pcDNA3.1-ATF6. Western blot analysis showed that the expression level of ATF6 protein in the ASP + ATF6 group was significantly higher than that in the ASP + vector group (*P* < 0.01, Fig. 5A, B). Therefore, ATF6 in CAFs was successfully over-expressed by transfection. In addition, compared with the ASP + vector group, the protein expression level of α-SMA in the ASP + ATF6 group increased significantly (*P* < 0.01, Fig. 5A, B). Collagen gel contraction and Transwell experiments showed that the contraction and migration ability of CAFs in the ASP + ATF6 group were significantly higher than those in the ASP + vector group (*P* < 0.01, Fig. 5C, D). Furthermore, RT-qPCR was conducted to evaluate the change in cytokine expression level. The results showed that the up-regulation of ATF6 reversed the inhibitory effect of ASP on the expression levels of IL-6, CCL2, and CXCL10 in CAFs (Fig. 5E–G). The above results indicated that the up-regulation of ATF6 reversed the inhibitory effect of ASP on the activation of CAFs.

## Discussion

Due to early invasive growth and high metastasis rate, pancreatic cancer is still a highly fatal malignant disease, and only about 10–20% of pancreatic cancer can be resected at diagnosis [23]. Pancreatic CAFs can regulate tumor progression by secreting growth factors and inflammatory mediators [9, 24, 25]. In this study, we detected the expression levels of fibroblast markers α-SMA, FAP, and Vimentin in CAFs and NFs. Notably, α-SMA is a marker of fibroblast activation, which is usually expressed in CAFs rather than in normal static fibroblasts. FAP is lowly expressed in most healthy adult tissues, and is mainly expressed in interstitial CAFs. Vimentin is a biomarker for EMT maintaining structure and movement during cell migration [26]. In this paper, the expression levels of α-SMA, FAP, and Vimentin in CAFs were significantly higher than those in NFs.

ASP is an iridoid compound with antioxidant, anti-tumor, and anti-inflammatory activities [17]. The anti-tumor effect of ASP has been reported in some studies. For example, ASP significantly inhibited the symptoms of colitis, and the number and size of tumors, and suppressed the progression of colorectal cancer [19]. Besides, ASP inhibits metastasis and angiogenesis by regulating VEGF, DII4, Notch, and Ang1/Ang2 signaling pathways, thereby inhibiting breast cancer [27]. In this study, ASP could inhibit the activation of CAFs in pancreatic cancer and reduce the contraction and migration ability of cells, and its anti-tumor effect is consistent with previous reports.

CAFs can regulate tumor microenvironment by releasing cytokines (IL-6) and chemokines (CCL2 and CXCL10). These cytokines and chemokines can retain the inhibitory immune subsets and hinder the normal function of cytotoxic lymphocytes, or form a physical barrier through ECM remodeling to prevent immune cells from entering [4]. Previous studies reported the effect of ASP on cytokines. Specifically, ASP treatment can not only reduce the levels of TNF-α, IL-1β, IL-6, and RANKL in the tissues around the implant [28], but also reduce the levels of TNF-α, IL-1β and IL-6 in RAW 264.7 cells stimulated by lipopolysaccharide [29]. In this study, ASP treatment decreased the expression levels of *IL-6*, *CCL2,* and *CXCL10* in CAFs.

ATF6 is a stress sensor located in the ER. The expression level of ATF6 was highly expressed in the activation of CAFs, and was regulated by Nrf2-p62 [13]. In addition, the increase in ATF6 expression was related to the increase of apoptosis, ER, and mitochondrial diseases in pancreatic tissues of patients with acute pancreatitis and PRSS1 mice [30]. In this study, ASP treatment reduced the expression of ATF6 in CAFs. Subsequently, we discovered that over-expression of ATF6 could alleviate the inhibitory effect of ASP on the contraction and migration ability of CAFs. Previous studies have pointed out that ASP can up-regulate the expression of ATF6 in acute myeloid leukemia cells U937 and HL-60, activate ER stress, and induce apoptosis [21]. This is contrary to our results, which may be related to the difference in disease types and cell lines. Furthermore, ATF6 is the key signal of ER stress. ATF6 can activate ER stress and unfolded protein reaction, and induce apoptosis by regulating the expression of pro-apoptotic molecules [31]. We suspect that the inhibitory effect of ASP on pancreatic CAFs may be related to ER stress, but further investigation is needed in subsequent research.

There are still some limitations in this study. Firstly, related animal experiments were not conducted to verify the effectiveness of ASP in pancreatic cancer. Secondly, the downstream mechanism of ATF6 regulating the activation of CAFs was not further explored. Therefore, it is necessary to verify the anti-tumor effect and molecular mechanism of ASP through animal experiments in the future.

## Conclusion

To sum up, ASP inhibits the activation of pancreatic CAFs by down-regulating the expression of ATF6, and reduces the contraction and migration ability of CAFs in a concentration-dependent manner. Our research shows that ASP has a certain application prospect in the treatment of pancreatic cancer.

### Supplementary Information

Supplementary material 1.

Supplementary material 2.

## Data Availability

Datasets used in this article are available from the corresponding author on reasonable request.

## Abbreviations

ATF6: Activating transcription factor 6
ASP: Asperuloside
BSA: Bovine serum albumin
CAFs: Cancer-associated fibroblasts
CCL: C–C motif chemokine ligand
CXCL: C-X-C motif chemokine ligand
DSS: Disease-specific survival
DMEM: Dulbecco’s modified eagle medium
ER: Endoplasmic reticulum
ECM: Extracellular matrix
FBS: Fetal bovine serum
FAP-1: Fibroblast activating protein 1
GAPDH: Glyceraldehyde 3-phosphate dehydrogenase
MAPK: Mitogen-activated protein kinase
NFs: Normal fibroblasts
OS: Overall survival
PBS: Phosphate-buffered saline
RIPA: Radio immunoprecipitation assay
RFS: Recurrence-free survival
RT-qPCR: Reverse transcription-quantitative polymerase chain reaction
TBST: Tris-buffered saline with Tween
VDR: Vitamin D receptor
α-SMA: α-Smooth muscle actin
