# Supplementary material for: Asperuloside inhibits the activation of pancreatic cancer-associated fibroblasts via activating transcription factor 6
Source: Discov Oncol. 2024 Jun 19;15:234. doi: 10.1007/s12672-024-01095-w (PMC11187058; doi:10.1007/s12672-024-01095-w)
Supplement: Supplementary file 1 — Supplementary material 1. [file 12672_2024_1095_MOESM1_ESM.pdf]

Figure 2B

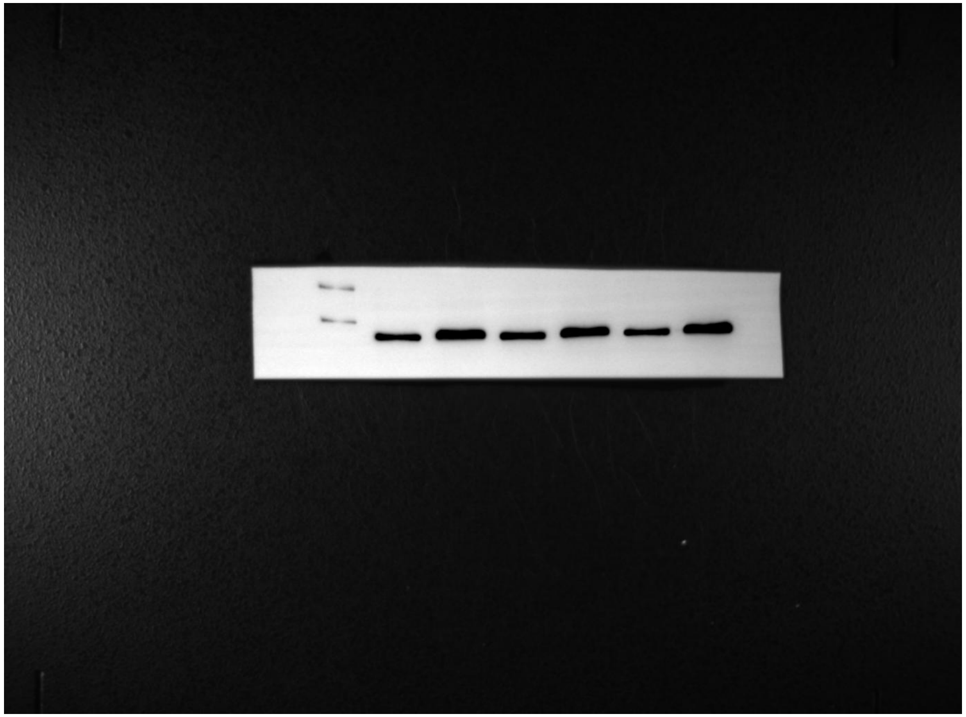

The original Western Blot images of α-SMA in Figure 2B. From left to right: 1# NFs, 1# CAFs, 2# NFs, 2# CAFs, 3# NFs, 3# CAFs groups

Figure 2B

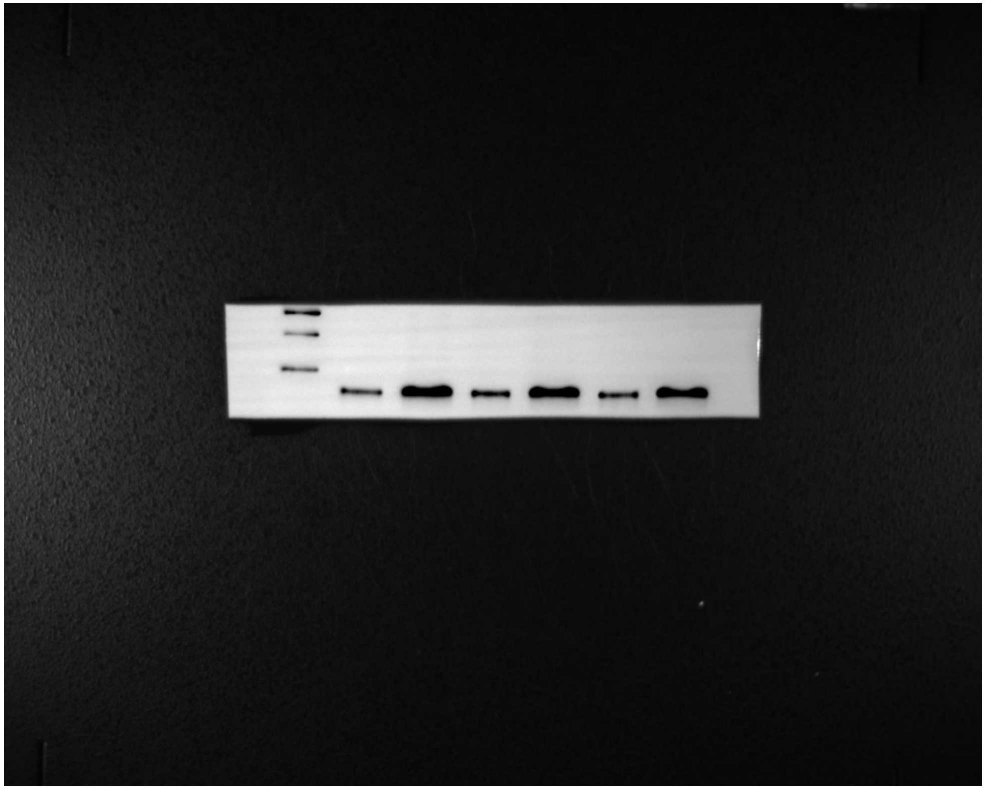

The original Western Blot images of FAP in Figure 2B. From left to right: 1# NFs, 1# CAFs, 2# NFs, 2# CAFs, 3# NFs, 3# CAFs groups

Figure 2B

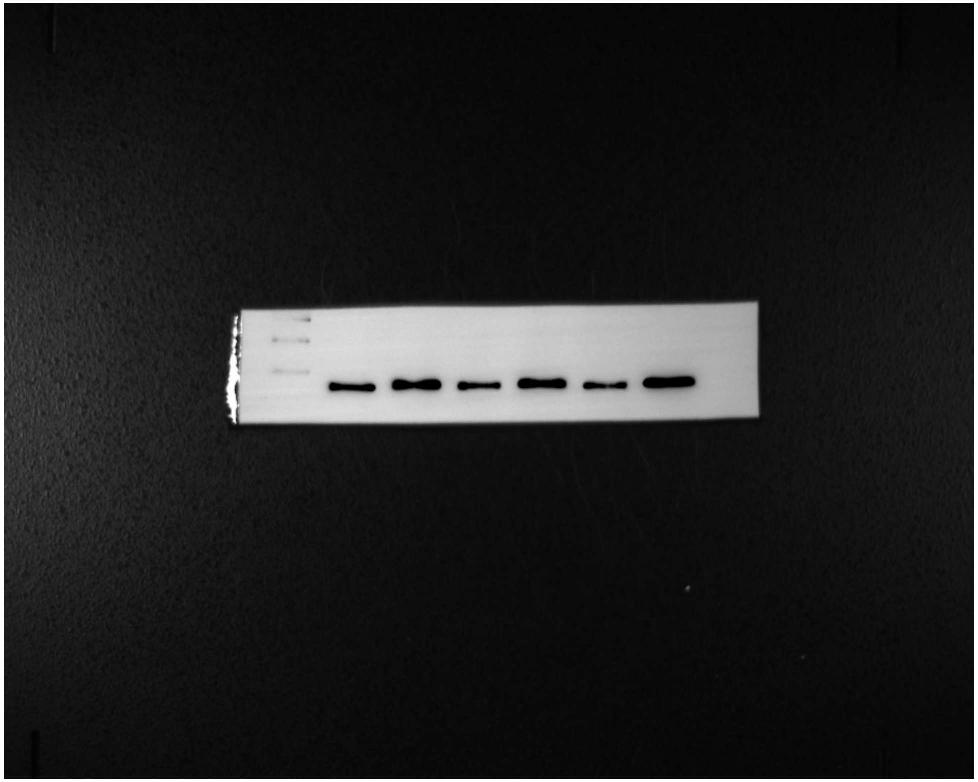

The original Western Blot images of Vimentin in Figure 2B. From left to right: 1# NFs, 1# CAFs, 2# NFs, 2# CAFs, 3# NFs, 3# CAFs groups

Figure 2B

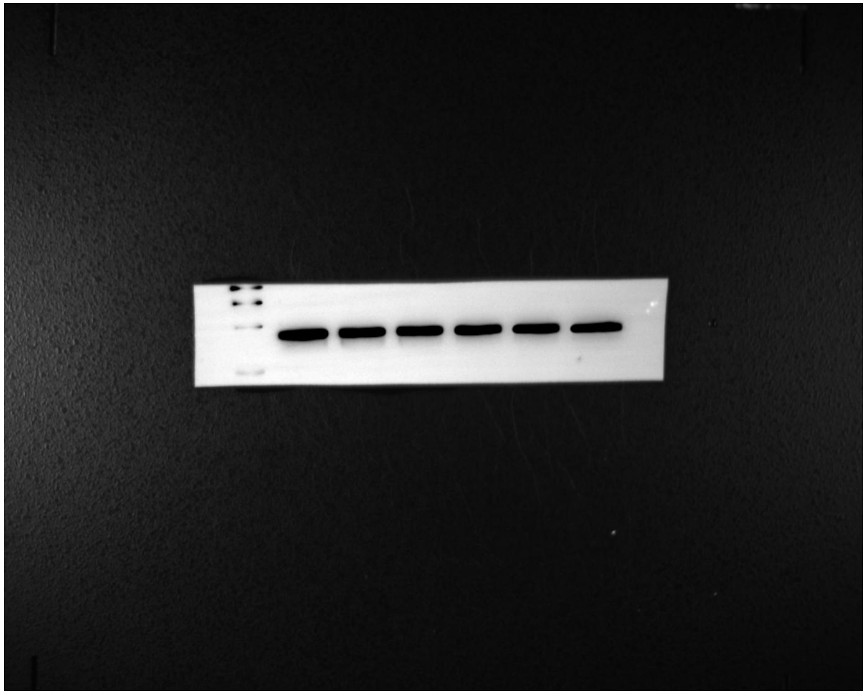

The original Western Blot images of GAPDH in Figure 2B. From left to right: 1# NFs, 1# CAFs, 2# NFs, 2# CAFs, 3# NFs, 3# CAFs groups

Figure 3B

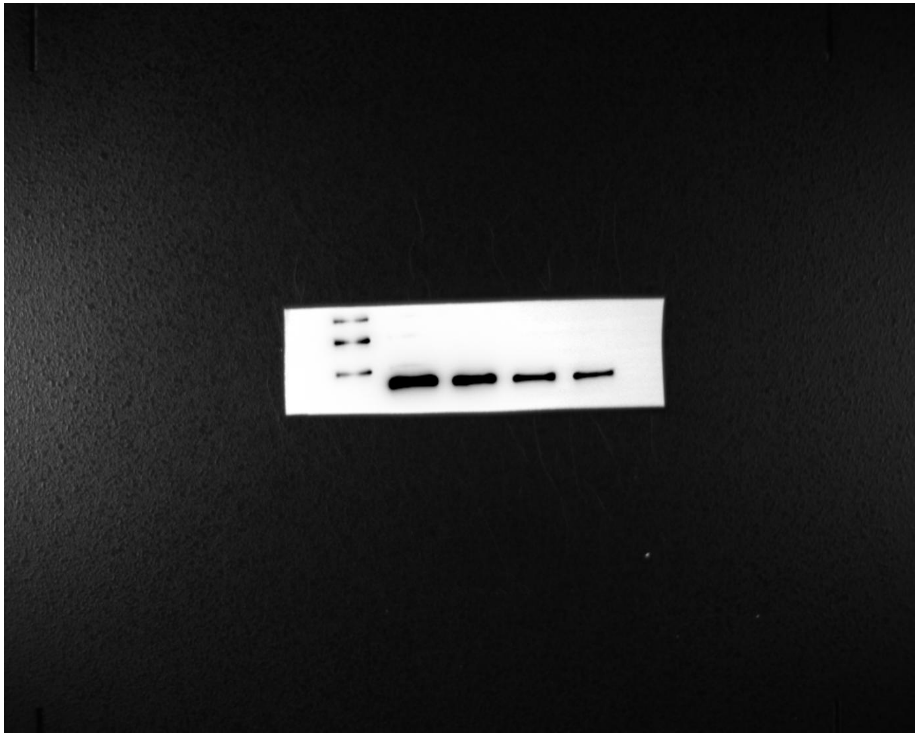

The original Western Blot images of α-SMA in Figure 3B. From left to right: 0 mM, 1 mM, 3 mM, 5 mM groups.

Figure 3B

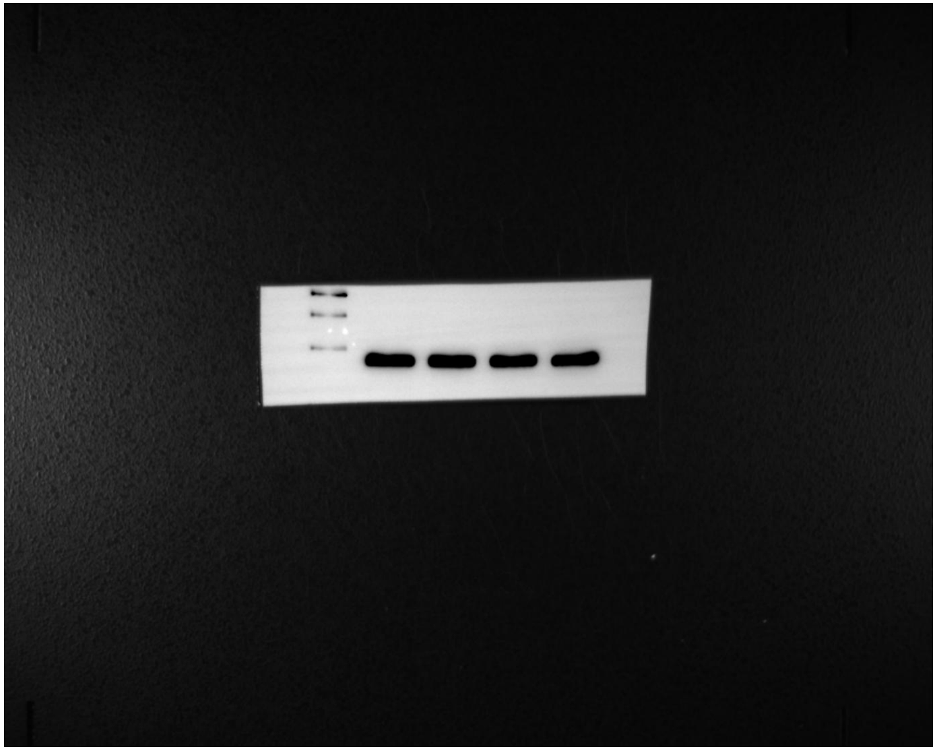

The original Western Blot images of GAPDH in Figure 3B. From left to right: 0 mM, 1 mM, 3 mM, 5 mM groups.

Figure 4

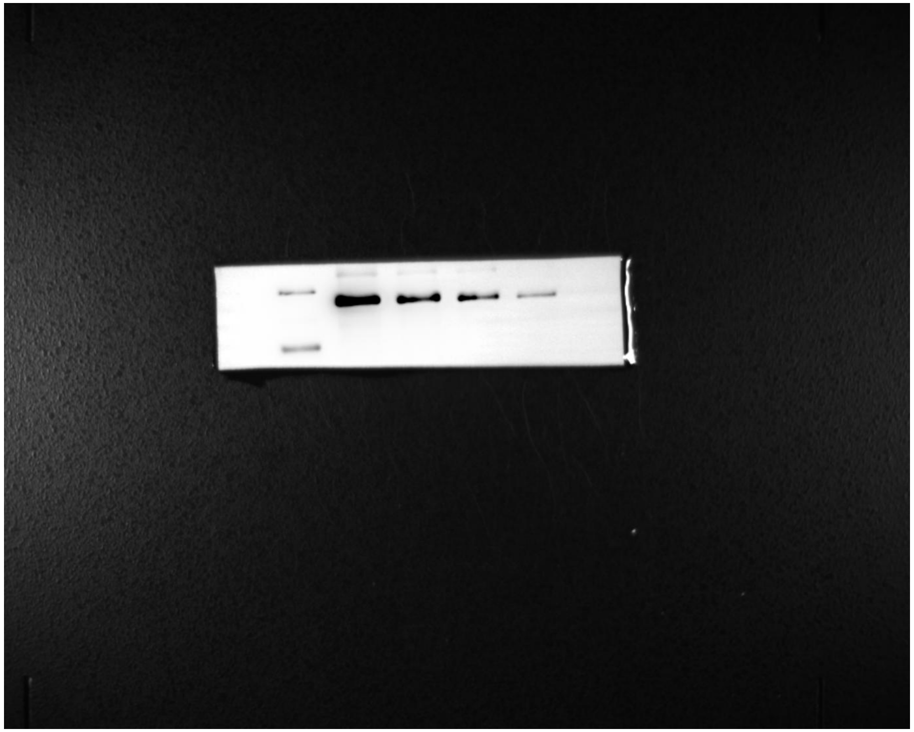

The original Western Blot images of ATF6 in Figure 4. From left to right: 0 mM, 1 mM, 3 mM, 5 mM groups.

Figure 4

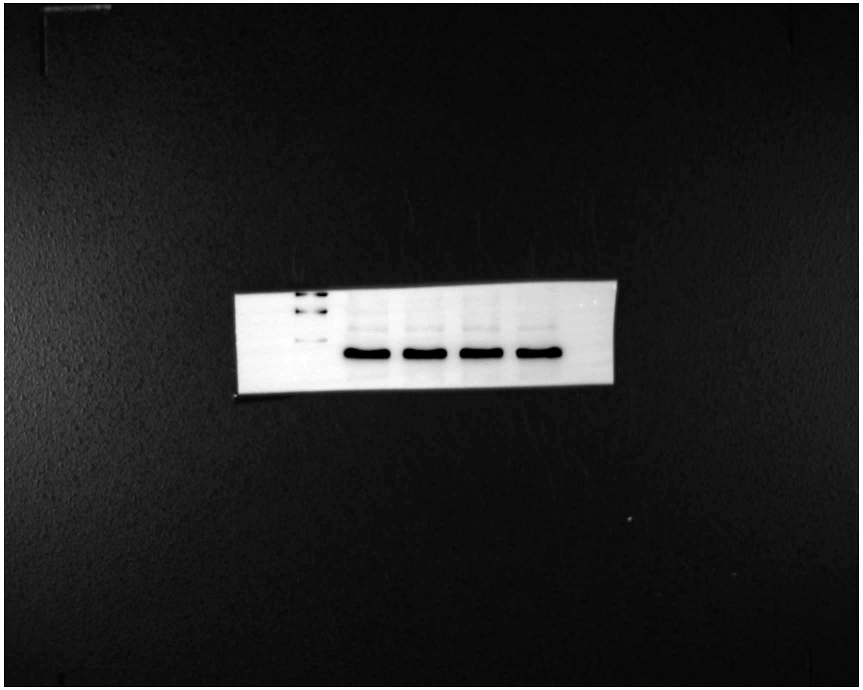

The original Western Blot images of GAPDH in Figure 4. From left to right: 0 mM, 1 mM, 3 mM, 5 mM groups.

Figure 5A

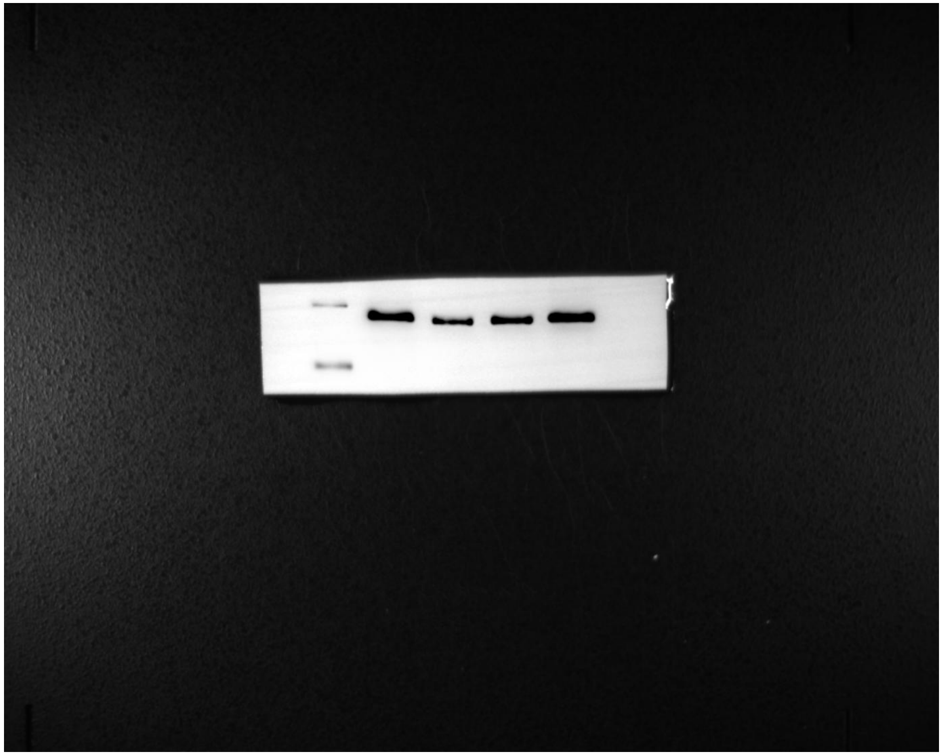

The original Western Blot images of ATF6 in Figure 5A. From left to right: Control, ASP, ASP+vector, ASP+ATF6 groups.

Figure 5A

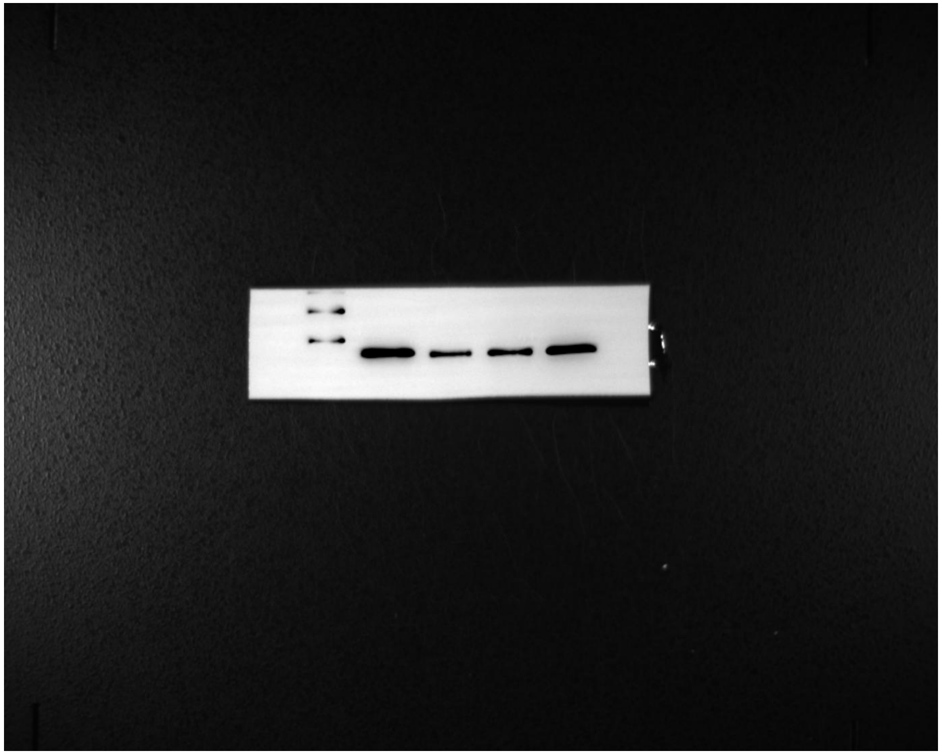

The original Western Blot images of α-SMA in Figure 5A. From left to right: Control, ASP, ASP+vector, ASP+ATF6 groups.

Figure 5A

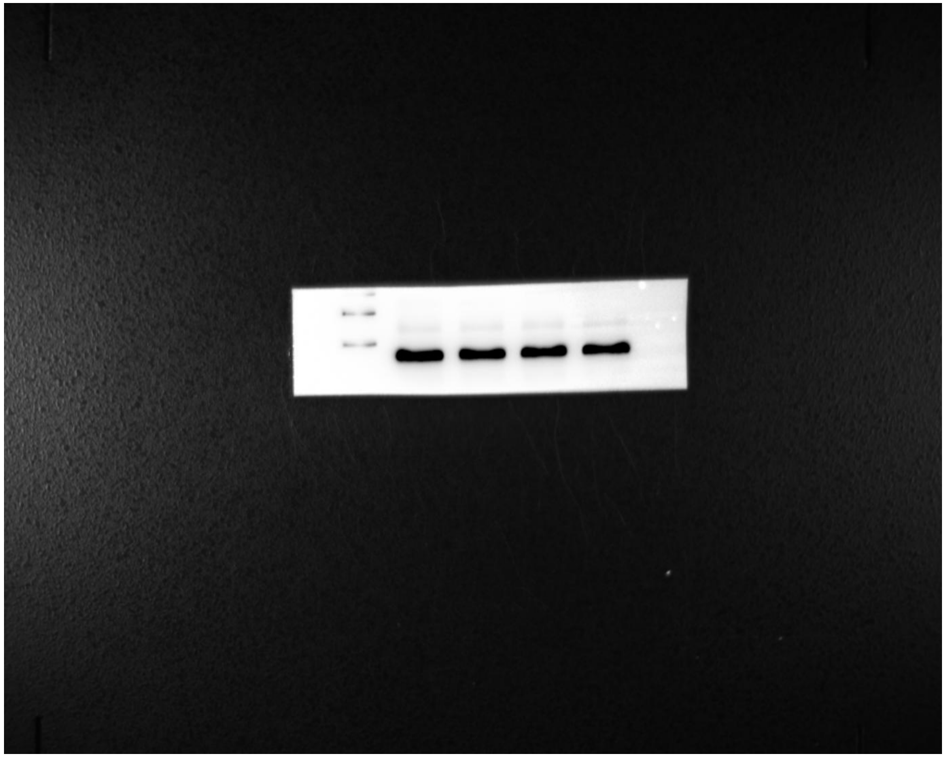

The original Western Blot images of GAPDH in Figure 5A. From left to right: Control, ASP, ASP+vector, ASP+ATF6 groups.
